# Uncommon Cause, Critical Consequence: Vertebral Artery Aneurysm Resulting in Intraventricular Hemorrhage

**DOI:** 10.7759/cureus.63179

**Published:** 2024-06-26

**Authors:** Roma Chavhan, Sourya Acharya, Anil Wanjari, Nitish Batra, Nishant Rathod

**Affiliations:** 1 General Medicine, Jawaharlal Nehru Medical College, Datta Meghe Institute of Higher Education and Research, Wardha, IND

**Keywords:** posterior inferior cerebellar artery, vertebral artery dissection, intraventricular haemorrhage, subarachnoid hemorrhage, vertebral artery aneurysm

## Abstract

The vertebrobasilar system is made up of the two vertebral arteries that unite to form the basilar artery near the base of the skull. Aneurysms in the vertebrobasilar system are distinct from other cerebral aneurysms due to their unique morphologic characteristics. They can be large and bulging (massive), pressing on the brainstem. Alternatively, they might be weak and splitting apart (dissecting) or have an elongated shape (fusiform). On the other end of the spectrum, Vertebral artery aneurysms (VAAs) can also be small and rounded (saccular). These aneurysms can occur at the vertebral artery itself or where it joins the posterior inferior cerebellar artery (PICA). Anatomically, they are situated near the brainstem and cranial nerves, deep within the posterior fossa. The cerebrospinal fluid is kept in transit and flux by the ventricular system's chambers circulating the fluid within themselves. An intraventricular hemorrhage (IVH) can occasionally result from vertebral artery aneurysmal ruptures that result in bleeding into the subarachnoid space and then extravasate into the ventricles. Persistent and poorly controlled hypertension affects about 50% of individuals with IVH. In this case report, we study a 74-year-old woman who complained of a sudden onset headache that had been bothering her for three days at the medical emergency room. She had been diagnosed with systemic hypertension eight years prior and had not taken her medicines as prescribed. She was discovered to have rigidity in her neck and a blood pressure reading of 170/100 mmHg, which had been followed by an episode of vomiting. Radiological investigations revealed a VAA that had a high risk of rupturing and causing an IVH.

## Introduction

The "Circle of Willis," a hexagon-shaped vascular network connecting the anterior and posterior brain circulations, is formed by the basilar and internal carotid arteries, which anastomose with one another near the base of the brain. This reflects the importance of the vertebral artery for humans [1]. An aneurysm is an attenuated yet bulging area in the wall of a blood vessel, resulting in an abnormal widening or ballooning greater than 50% of the vessel's normal diameter [2]. Vertebral artery aneurysms (VAAs) are rare, accounting for only 3% of intracranial aneurysms. They are frequently discovered in the third and fifth decades of life and are primarily linked to hypertension [3]. Individuals get intense headaches that start suddenly. There are additional signs of meningism such as photophobia, nausea, vomiting, and stiffness in the neck. It is vitally important to identify these aneurysms since larger hemorrhages can cause loss of consciousness, seizures, and brainstem compression with cardiorespiratory failure [4]. They can present with subarachnoid hemorrhage (SAH), medullary compression, and cranial neuropathies. Three percent of intracranial aneurysms are caused by dissecting aneurysms of the intradural vertebral artery, which is an infrequent source of SAH. If not secured right away, it is linked to a significant risk of rupture and a high death rate [5].

## Case presentation

A 74-year-old woman came to the emergency department with a headache that began in the occipital region, acute in onset and constant in nature. The headache was graded 7 on the pain scale and was associated with neck pain and one episode of vomiting. The patient also complained of dizziness, which was a sudden onset with no improvement in rest. There was no history of recent trauma or falls. The history of the patient included systemic hypertension and irregular medication. General examination was suggestive of the patient's heart rate of 130 beats per minute, decreased respiratory cycles (13/minute), and a blood pressure reading of 170/100 mmHg. The patient was examined in the supine position. She was arousable to tactile stimuli, had a short attention span, and required repeated instructions, but was oriented to time, place, and person. All routine laboratory investigations were done, as shown in Table 1.

**Table 1:** Laboratory parameters of the patient on admission INR - international normalized ratio

Lab parameters	Observed value	Normal range
Hemoglobin	11.8 g/dL	11-16 g/dL
Total leucocyte counts	14,500/cumm	4,000-11,000/cumm
Platelets	1.69/cumm	150,000-450,000/cumm
Mean corpuscular volume	86 fL	83-101 fL
Urea	25 mg/dL	18-42 mg/dL
Creatinine	1.01 mg/dL	0.65-1.24 mg/dL
Potassium	4.7 mmol/L	3.5-5.2 mmol/L
Sodium	138 mmol/L	136-144 mmol/L
Aspartate aminotransferase	32 U/L	14-57 U/L
Alkaline phosphatase	86 IU/L	37-125 IU/L
Alanine aminotransferase	38 U/L	<35 U/L
Total protein	7.5 g/dL	6.2-8.3 g/dL
Total bilirubin	1.0 mg/dL	0.20-1.30 mg/dL
Random blood sugar	92 mg/dL	70-110 mg/dL
Serum calcium	8.2 mg/dL	8.2-10.20 mg/dL
Serum magnesium	1.8 mg/dL	1.60-2.30 mg/dL
Serum phosphorous	4.2 mg/dL	2.4-4.6 mg/dL
INR	1.0	0.8-1.2

**Figure 1:**
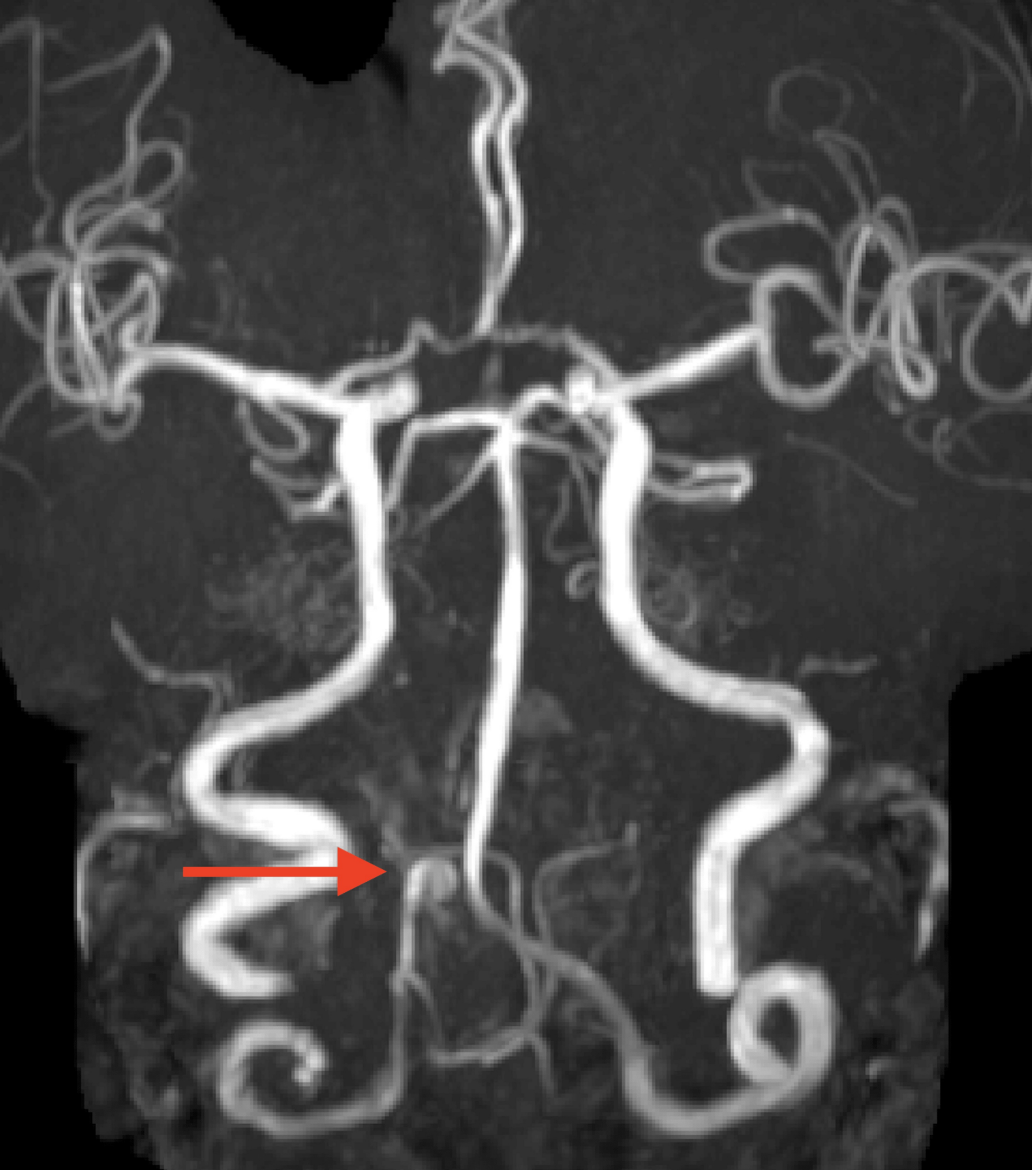
MR angiogram showing focal dilatation in the intracranial part of the right vertebral artery with a red arrow. MR - magnetic resonance

Evaluation of cranial nerves revealed no abnormal findings. The patient looked clumsy, a possible sign of muscle weakness, although there was no atrophy of musculature noted in any of the four limbs. Movements could be performed against gravity but not in the presence of resistance (power grade 3/5). The patient had a wide-based gait. Coordination testing with finger-to-nose and heel-to-sheen tests was carried out with much difficulty. Sensory examination was unremarkable. All deep tendon reflexes could be elicited, and there was a bilateral extensor plantar response. Rigidity was observed by passively flexing the neck. The patient's Glasgow Coma Scale (GCS) score was 13. However, her sluggish responses, coupled with a history of negligence of her anti-hypertensive medication, raised clinical suspicion, prompting immediate imaging and radiological intermediation. She underwent a magnetic resonance angiogram (MRA) as well as a venogram, which was suggestive of a focal dilatation in the intracranial part of the right vertebral artery in the pre-medullary cistern measuring approximately 4 × 6 mm with a narrow neck indicating an aneurysm as shown in Figure 1.

**Figure 2:**
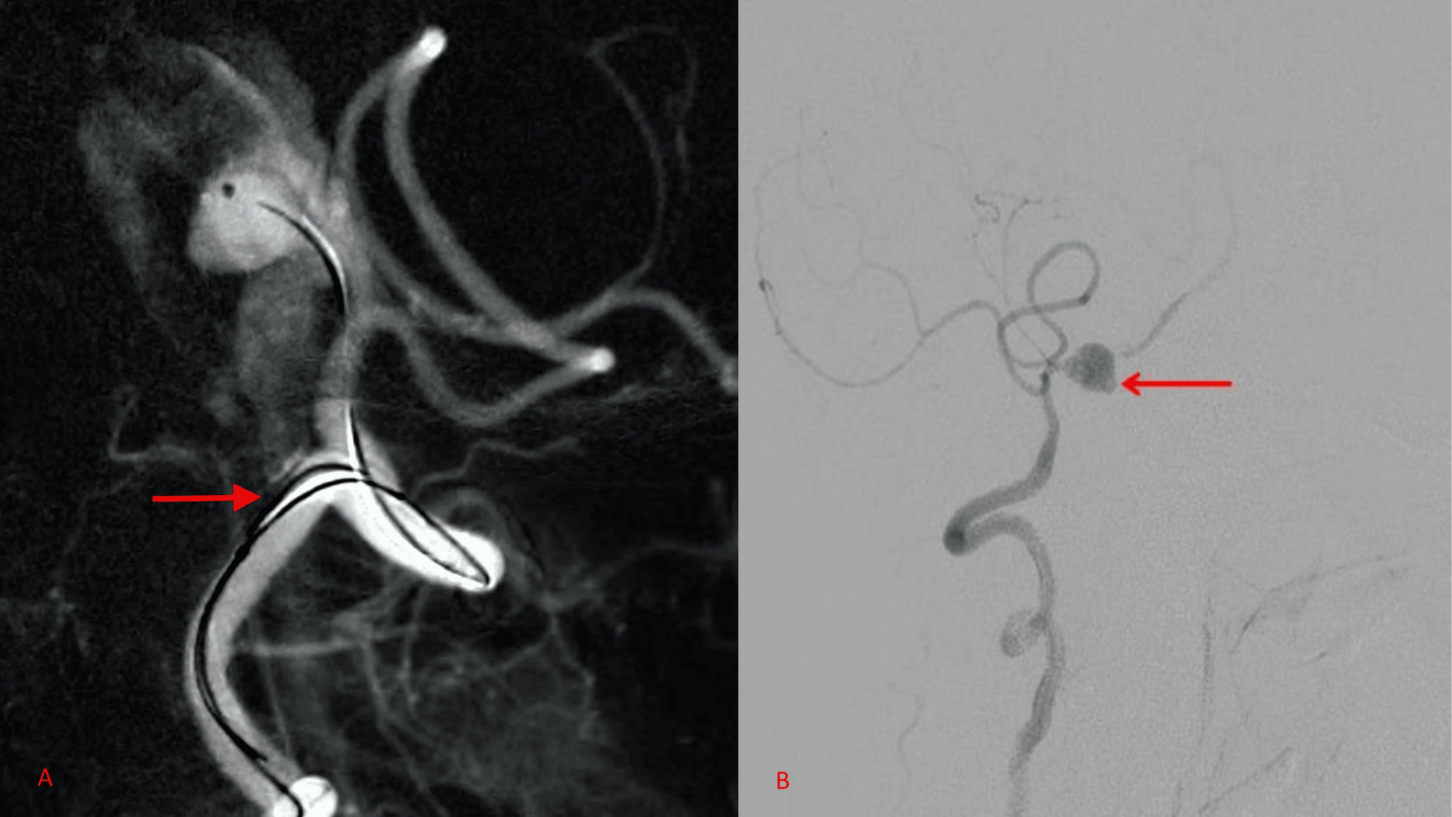
Digital subtraction angiogram (DSA) with a red arrow, showing a right vertebral artery V4 segment dissecting aneurysm. A) Lateral view; B) oblique view.

The possibility of aneurysmal rupture could not be excluded; hence, the patient was posted for a four-vessel digital subtraction angiogram (DSA) and endovascular coiling. With the patient under local anesthesia, a 5F Picard catheter was advanced into her right aortic arch, following which a right vertebral artery V4 segment dissecting aneurysm was noted, as shown in Figures 2A-2B.

**Figure 3:**
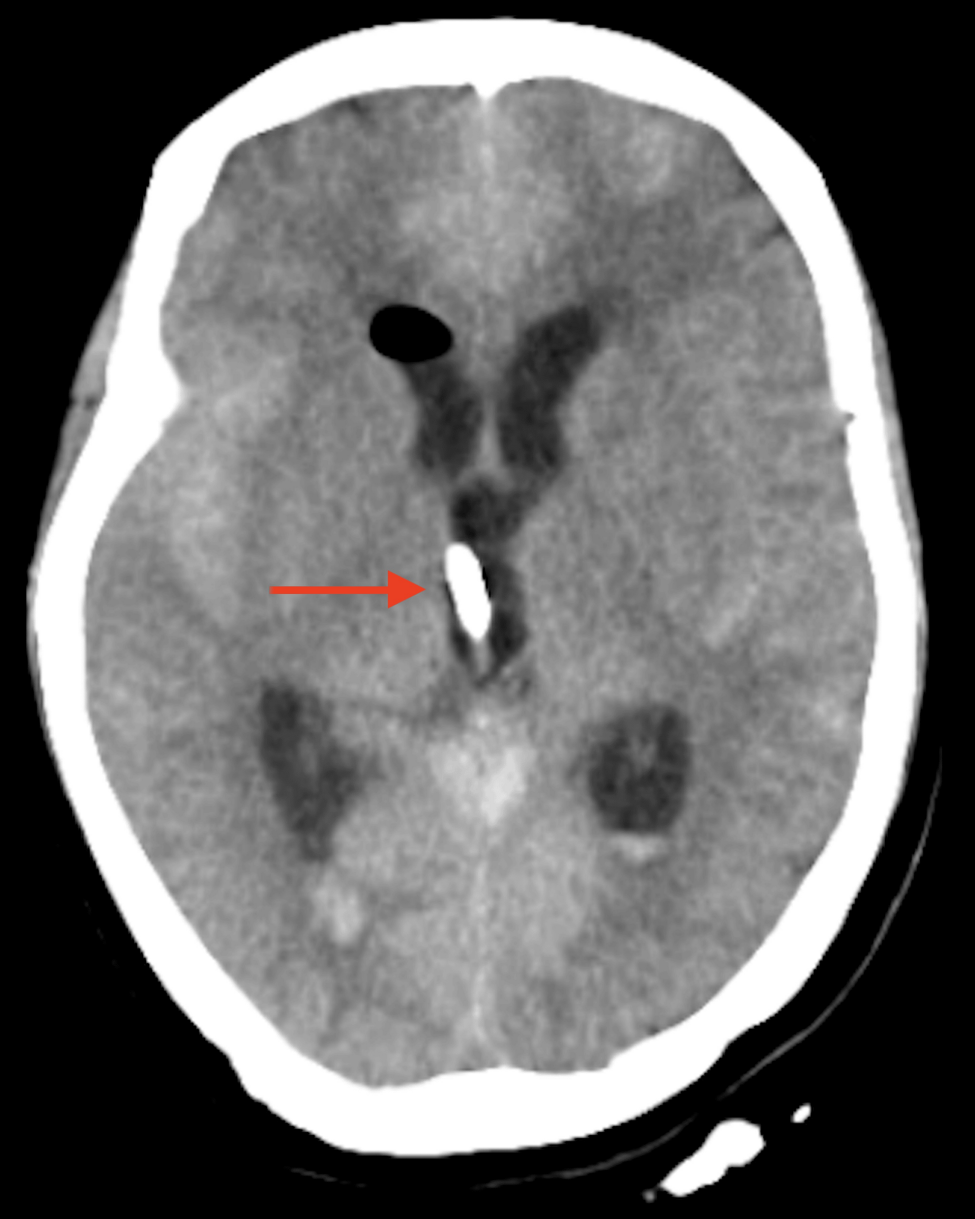
CT brain showing intraventricular hemorrhage in the third ventricle with red arrow along with subarachnoid hemorrhage.

Thereafter, the patient was supposed to be planned for end-vascular coiling of the parent vessel. At the time of the procedure, the patient's blood pressure shot up to 200/100 mmHg, and the patient sustained an aneurysmal SAH (aSAH). The aSAH also produced an intraventricular hemorrhage (IVH) as it oozed into the patient's third ventricle and the occipital horn of the right lateral ventricle, as shown in Figure 3.

**Figure 4:**
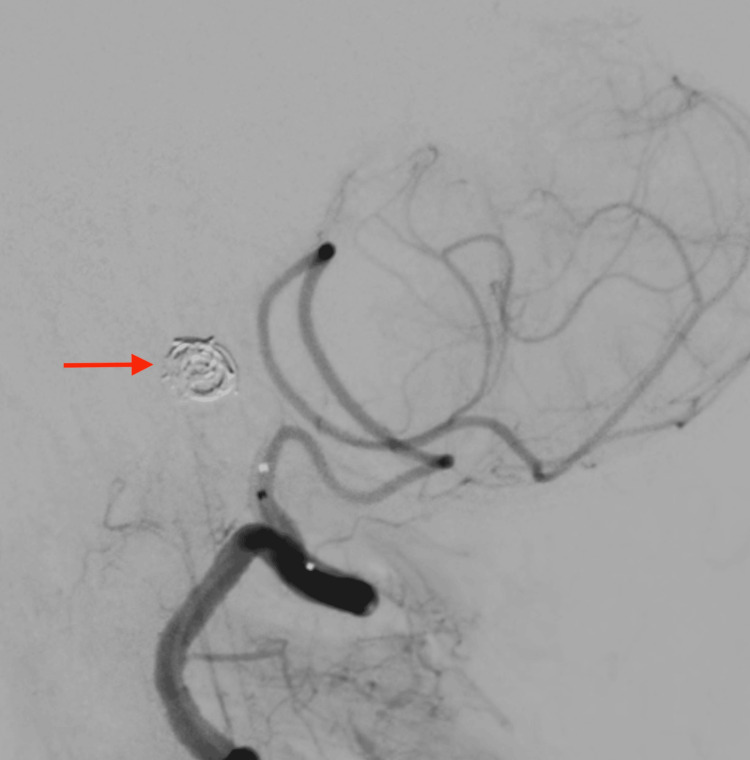
Red arrow showing endovascular coiling for right vertebral artery V4 segment dissecting aneurysm.

Intraoperatively, the patient had tachypnea, tachycardia, and an increase in blood pressure. Given her worsening GCS, she was put on invasive mechanical ventilation. Under general anesthesia, the patient was taken for coiling of the aneurysm. Once it was visualized, a microcatheter and Traxcess 014 microwire were used to catheterize the aneurysm, and it was coiled with a single detachable coil, causing occlusion of the vessel, as shown in Figure 4.

An emergency neurosurgery opinion was taken for the insertion of an external ventricular drain (EVD) to tackle the aSAH. EVD was inserted to normalize the flow of cerebrospinal fluid. It is one of the most important emergency procedures in neurosurgery, and it is mainly used to monitor intracranial pressure (ICP), as increased ICP should be normalized as soon as possible. Serial brain imaging was done after the insertion of an EVD, and the patient was eventually weaned off mechanical ventilation. Timely interventions were responsible for altering the patient's status and yielded a favorable outcome.

## Discussion

A rare cause of SAH, accounting for 3% of all cerebral aneurysms, is a dissecting aneurysm of the intradural vertebral artery [5].

Less than 5% of all aneurysms are VAAs, making them extremely uncommon. VAA are aneurysms that usually develop intracranially where the posterior inferior cerebellar artery (PICA) and vertebral artery meet, which account for 0.5-3% of intracranial aneurysms. VAD leads to SAH. In Japan, in a nationwide study, it has been reported that bleeding and ischemia occur in 30.5% to 33.1% of cases [6]. The surrounding clinical anatomy of the vertebral arteries makes the treatment of an aneurysm a daunting task for the clinician as well as a neurosurgeon. The medulla, the tonsils, the inferior vermis, the choroid plexus, the tela choroidea of the fourth ventricle, and the inferior aspects of the cerebellar hemispheres are all supplied by the PICA. PICA syndrome (posterior cerebellar artery syndrome) is also a possible complication and may cause slurred speech, diplopia, dizziness, and Horner syndrome [7]. Cerebellar and brain stem infarction, and SAH are also possible complications. SAH and CNS compression neuropathy have been reported in young patients due to COVID-19 [8]. Before symptoms appear, patients usually report little, generally harmless triggering events. VAD is a silent transgressor as the clinician is likely to think of the headache as migraine, especially in young adults. Half of the cases of intracranial dissection involve a SAH. Compared to patients with extracranial dissections, this group of patients has a far worse prognosis and significant neurological symptoms. Studies show that people with VAD are, more often than not, asymptomatic and remain undiagnosed. Modifying the vertebrobasilar circulation's hemodynamics could result in unpredictable long-term consequences. It is imperative to diagnose the condition as soon as possible to prevent stroke, which may occur within several days or weeks of VAD or even result in serious long-term sequelae [6]. The first investigations that are obtained frequently involve CT and CT angiography (CTA). In addition to showing SAH or posterior fossa ischemia, CT can show an obstructed vertebral (hyperdense) or mural thrombus (thickened wall, frequently with some surrounding stranding). There may occasionally be a recognizable "double lumen" look on CT. MRI is more sensitive at imaging intramural hemorrhage in addition to having a significantly greater sensitivity to small foci of ischemia (using diffusion-weighted imaging (DWI)) and the capacity to scan the vascular lumen (MRA). Conventional angiography is traditionally considered the gold standard. It could show fusiform aneurysmal dilatation, proximal or distal stenosis, or localized dilatation. Whether or not the dissection extends into the intracranial compartment has a significant impact on the prognosis and course of treatment. If the latter is accurate, SAH is common and typically has catastrophic results [9]. Headache may be regarded as an acute phase biological surrogate linked to early dissecting aneurysm rupture and dynamic arterial alterations in patients with acute VAD [10]. The two main types of spontaneous VAD are as follows: 1) the ischemic type, which is characterized by ischemia symptoms and/or vertebrobasilar circulation infarction as a result of thromboembolism and arterial narrowing, and 2) the hemorrhagic type, which manifests as a SAH brought on by the rupture of an intradural vertebral artery dissecting aneurysm. SAH is linked to over 50% of intracranial VADs. Intracerebral dissections typically result in significant neurological impairments or SAH and have a dismal prognosis.

All patients with acute dissections of the vertebral artery, regardless of the type of symptoms, have been advised to undergo anticoagulation with intravenous heparin followed by oral warfarin to prevent thromboembolic complications, unless there are contraindications such as the presence of a large infarct with associated mass effect, hemorrhagic transformation of the infarcted area, an intracranial aneurysm, or intracranial extension of the dissection. Most VADs recover on their own. However, in patients who present with SAH, an immediate surgical intervention can be necessary. Surgery has mostly been replaced by endovascular therapy as the first line of treatment when medical therapy is ineffective or not appropriate [11].

Microsurgical clip obliteration of intracranial aneurysms was the primary modality of treatment and the first reported treatment alternative before 1991. Technological advancement has led to widespread acceptance of endovascular treatment of posterior circulation aneurysms. The risk of long-term consequences was 5.4%, and the death rate from coiling a basilar bifurcation aneurysm was 0.9%, according to a meta-analysis. Endovascular coiling should be taken into consideration for patients with ruptured aneurysms who are deemed technically suitable for both neurosurgical clipping and endovascular coiling. Of those with aSAH, 15% to 87% develop acute hydrocephalus. The management of acute hydrocephalus linked to aSAH often involves either lumbar drainage or EVD. Neurological improvement is frequently related to EVD for patients with aSAH-associated hydrocephalus. Following the placement of an EVD, the was a significant improvement in the patient's GCS score, and she was weaned off invasive ventilation three days later. The course of therapy for individuals with burst aneurysms also seems to affect the emergence of seizures later on. Research involving patients undergoing endovascular therapy revealed a 3% incidence of delayed seizures and no periprocedural seizures. In the early posthemorrhagic phase, preventive anticonvulsant treatment may be taken into consideration [12].

## Conclusions

An interdisciplinary team of a radiologist, neurologist, and interventional neurologist, interventional radiologist is ideally suited to handle the complex diagnosis and treatment of vertebral artery dissection. Following the procedure, the patient experienced a complete resolution of her headaches. Repeat imaging demonstrated stable occlusion of the aneurysm with preserved vertebral artery flow. This case report aims to highlight the complications of VAAs, such as brainstem infarctions and pseudoaneurysms, leading to compression neuropathy of the cranial nerves and stroke if not identified and treated immediately. This case underscores the importance of considering vascular etiologies, including VAAs, in patients presenting with headaches refractory to conventional therapy. Prompt recognition and appropriate management can lead to favorable outcomes and prevent potentially life-threatening complications.
